# Fully automatic and robust 3D registration of serial-section microscopic images

**DOI:** 10.1038/srep15051

**Published:** 2015-10-09

**Authors:** Ching-Wei Wang, Eric Budiman Gosno, Yen-Sheng Li

**Affiliations:** 1 Graduate Institute of Biomedical Engineering, National Taiwan University of Science and Technology, Taipei city, Taiwan; 2 Department of Biomedical Engineering, National Defence Medical Center, Taiwan

## Abstract

Robust and fully automatic 3D registration of serial-section microscopic images is critical for detailed anatomical reconstruction of large biological specimens, such as reconstructions of dense neuronal tissues or 3D histology reconstruction to gain new structural insights. However, robust and fully automatic 3D image registration for biological data is difficult due to complex deformations, unbalanced staining and variations on data appearance. This study presents a fully automatic and robust 3D registration technique for microscopic image reconstruction, and we demonstrate our method on two ssTEM datasets of drosophila brain neural tissues, serial confocal laser scanning microscopic images of a drosophila brain, serial histopathological images of renal cortical tissues and a synthetic test case. The results show that the presented fully automatic method is promising to reassemble continuous volumes and minimize artificial deformations for all data and outperforms four state-of-the-art 3D registration techniques to consistently produce solid 3D reconstructed anatomies with less discontinuities and deformations.

Robust and fully automatic three-dimensional (3D) image registration of serial-section microscopic images is critical for detailed 3D anatomical reconstruction of large biological specimens such as serial section Transmission Electron Microscopy (ssTEM) of neural tissues^1^, serial confocal laser scanning microscopic images of a brain^2^,^3^ or serial histopathological microscopic images^4^,^5^. In biomedical applications, a large specimen is generally embedded in the medium block and cut into a series of microscopy image slices, called as sections, which are collected, stained and digitally imaged. The digital microscopic slices are then used to reconstruct detailed the 3D anatomy for further biological and medical investigation. Serial slides can be manually aligned by setting up a number of pairs of corresponding control points to the same (*x*, *y*) location for consecutive images *z*_*i*_ and *z*_*i*+1_, and the pairs of images and paired-sets of control points are then given to semi-automatic software^6^ for image alignment. Fully automatic registration of biological images is possible as demonstrated by the software - TrakEM2^1^,^7^,^8^,^9^ and in various studies^3^,^10^,^11^,^12^,^13^.

However, robust and fully automatic 3D registration of serial-section microscopic images is challenging as the disadvantage of serial-section microscopy is that cutting a block of specimen into several sections tends to create the discontinuity between every section and leads to deformation in individual sections^1^. Therefore, sections need to be aligned in order to remove deformation and discontinuity. Cardona *et al.*^8^ also pointed out that "TrakEM2 acknowledges that any automatic procedure (such as image registration and image segmentation) will eventually fail partially or fully and will require manual correction by a human operator". Moreover, in comparison to laser scanning confocal images as used in the studies^2^,^3^ where the serial image data maintains the property of geometrical continuity in 3D space, there are complex deformation problems for serial histopathological slides, including physical destructions caused by cutting and fixation, staining artifacts and uneven stain variations due to potential discrepancy in thickness of individual tissue sections. These complex distortion effects makes image registration of histopathological data an even harder task.

The main contribution of this study is to present a fully automatic and robust 3D image registration method for reconstruction of detailed 3D anatomy and able to deal with complex deformation problems for different types of microscopic images, such as serial ssTEM images, laser scanning confocal images and histopathological images. The proposed 3D image registration system contains a new 3D alignment and validation model utilizing the B-Spline Deformation Field and our recent efforts on robust 2D image registration^4^,^5^. Details are described in the methodology section. The experimental results show that the proposed fully automatic method is promising to perform 3D registration well for all data and consistently produces solid 3D reconstructed objects with less discontinuities and deformations in comparison to the benchmark methods.

## Results

Regarding the experimental materials, four serial-section microscopic image data sets are tested, including two sets of ssTEM images of the neural tissues of the droshophila brain^14^,^15^, containing 20 and 30 gray images respectively, a set of 18 serial histopathological color images of renal cortical tissues^4^, and a set of 108 serial laser scanning microscope images of the drosophila brain^2^. In addition to the real world serial-section microscopic image sets, a synthetic test case is built for quantitative evaluation. Regarding the benchmark approaches, four state-of-the-art 3D registration methods are compared with the proposed method using TrakEM2^1^,^7^,^8^,^9^, including a method using least squares (linear feature correspondence)^16^, an elastic b-spline model for biological images (UnwarpJ)^17^, an improved bi-directional b-spline model for histopathological section alignment (bUnwarpJ)^18^ and an elastic volume reconstruction method^1^, and the four benchmark registration methods are tested with four different transformation parameters, including translation, rigid, similarity and affine. Hence, there are 16 benchmark approaches tested in total.

**Figure 1:**
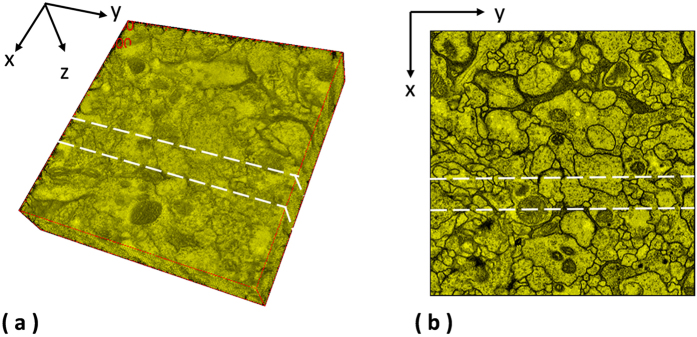
Extraction of side views for the ssTEM data^14^. Two planes are randomly chosen for the ssTEM data of the drosophila melanogaster third instar larva ventral nerve cord to extract the side views of the 3D reconstructed objects generated by individual methods.

In evaluation, for every data and method applied, registration results are reconstructed as a 3D anatomical object. In order to assess the continuity of the reconstructed 3D objects and evaluate the performance of the registration method, a randomly selected plane is defined for each dataset to extract side views of the 3D objects by individual methods. For example, in Fig. 1, (a) the inputs for 3D registration are original serial histopathological images. Without registration, (b) serial images are sequentially placed into a 3D space, and a randomly selected plane can be defined to cut the 3D object into two parts. Then, the side view of the upper part object can be used to assess the continuity of the reconstructed 3D object; without registration, the continuity of the reconstructed object is poor here. After registration by the proposed method, (c) registered images are sequentially placed in a 3D space to produce (d) a reconstructed 3D object. Next, the 3D object will be cut into two parts using the randomly selected plane defined previously, and the side view of the upper cut object is used to assess the continuity of the reconstructed anatomical object and evaluate the performance of the registration approach.

### Serial section Transmission Electron Microscopy (ssTEM) of Droshophila brain

**Figure 2:**
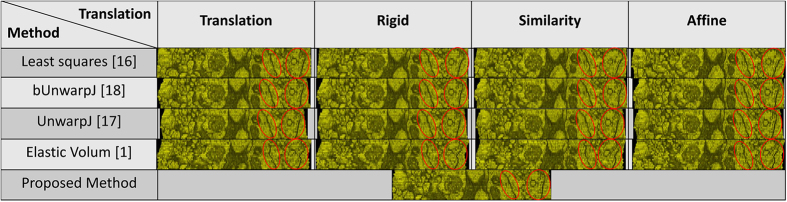
1st side views of the reconstructed anatomical objects for the ssTEM data^14^. Using the first plane chosen in Fig. 2, side views of the reconstructed anatomical objects by individual methods for the ssTEM data of the drosophila melanogaster third instar larva ventral nerve cord are displayed.

**Figure 3:**
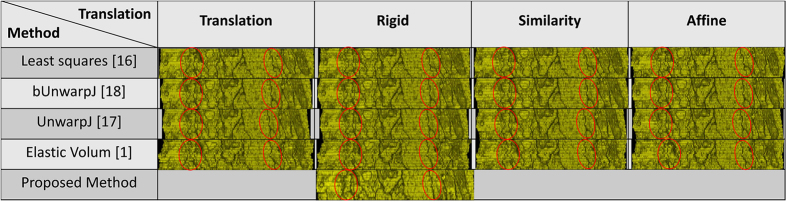
2nd side views of the reconstructed anatomical objects for the ssTEM data^14^. Using the second plane chosen in Fig. 2, side views of the reconstructed anatomical objects by individual methods for the ssTEM data of the drosophila melanogaster third instar larva ventral nerve cord are displayed.

Two ssTEM image sets of the droshophila brain^14^,^15^ are tested in the experiments. The first one is released by Gerhard *et al.*^14^, containing 20 sections from serial section Transmission Electron Microscopy (ssTEM) of the Drosophila melanogaster third instar larva ventral nerve cord (VNC), which were freshly dissected and collected from instar fly brain. Every image is in the dimension of 1024 by 1024 pixel, with a resolution of 4.6 × 4.6 nm/pixel. The cube measures 4.7 × 4.7 × 1 microns approx, with section thickness of 45–50 nm. For this dataset, two planes are randomly chosen as shown in the Fig. 1 to extract the side views of the 3D reconstructed objects generated by individual methods, and the side views of the reconstructed objects by individual methods are presented in the Figs 2 and 3 respectively, showing that the proposed method produces solid 3D reconstructed objects with less discontinuity and deformation problems in comparison to the benchmark methods. For illustration purposes, red circles are marked to show that the proposed method yields a good continuity while some benchmark methods generate discontinued contours.

**Figure 4:**
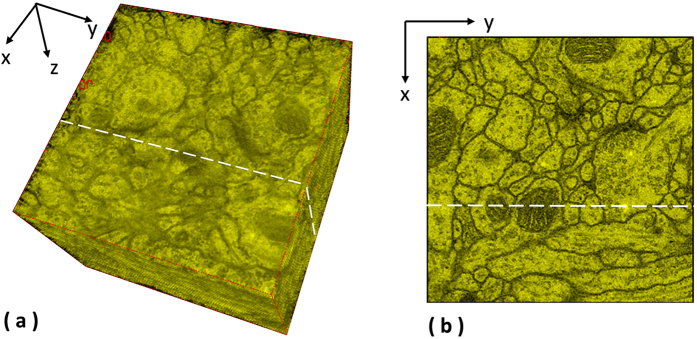
Extraction of side views for the ssTEM data^9^. One plane is randomly selected for the ssTEM data of the drosophila first instar larva brain neuropile and one ventral nerve cord segment to extract the side views of the 3D reconstructed objects generated by individual methods.

**Figure 5:**
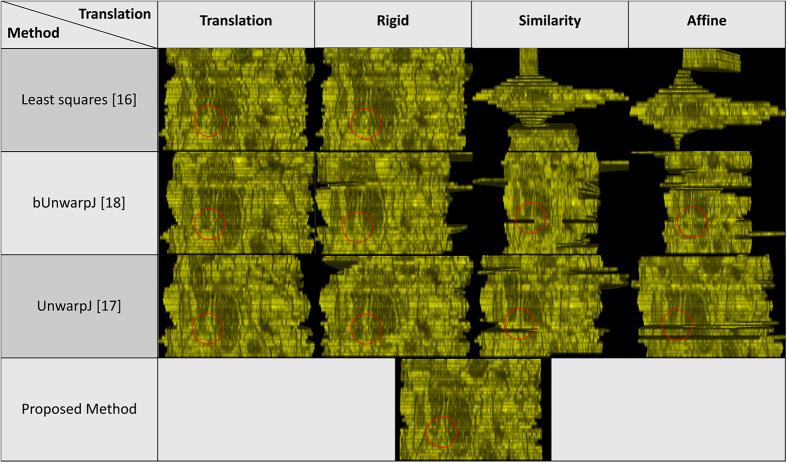
Side views of the reconstructed anatomical objects for the ssTEM data^9^. Side views of the reconstructed anatomical objects by individual methods for the ssTEM data of the drosophila first instar larval brain neuropile and one ventral nerve cord segment are displayed.

The second data set released by Cardona *et al.*^9^ contains 30 serial TEM sections of the drosophila first instar larval brain neuropile and one ventral nerve cord segment. Every image is in the dimension of 512 by 512 pixel, with resolution 4 nm/pixels and section thickness 50 nm. One plane as shown in Fig. 4 is randomly selected to extract the side views of reconstructed anatomical objects, and the side views of all objects are presented in Fig. 5, showing that the proposed method outperforms the benchmark approaches and yields a solid object with good continuity; a red circle highlights that the presented method align images well while the benchmark methods output more discontinuities. In addition, the fourth benchmark approach^1^ fails to find corresponding features among sections and is not able to generate registration outputs.

### Serial histopathological images of renal cortical tissues

**Figure 6:**
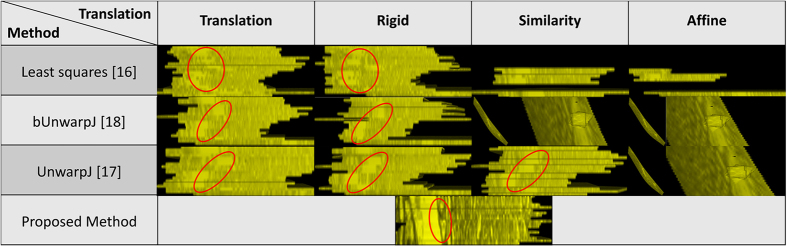
Side views of the reconstructed anatomical objects for the histopathological data^4^. Side views of the reconstructed anatomical objects by individual methods for the histopathological data are displayed.

**Figure 7:**
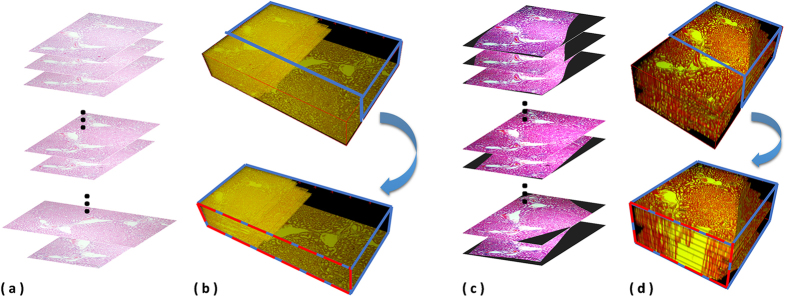
3D reconstruction using serial histopathological microscopic images. (**a**) the inputs for 3D registration are original serial histopathological images. Without registration, (**b**) serial images are sequentially placed into a 3D space, and a randomly selected plane can be defined to cut the 3D object into two parts. Then, the side view of the upper part object can be used to assess the continuity of the reconstructed 3D object. After registration by the proposed method, (**c**) registered images are sequentially placed in a 3D space to produce (**d**) a reconstructed 3D object.

18 serial histopathological images of renal cortical tissues^4^ are used as the third test data. This data is comparably more challenging due to large variations on the data appearances, image orientation and size among sections, complex deformations and artifacts introduced during the data preparation process, causing existing methods fail to reconstruct a solid 3D object and perform poorly as shown in the Fig. 6. In addition, the fourth benchmark approach^1^ fails to find corresponding features among sections and is not able to generate registration outputs. In comparison, the proposed method well aligns images in 3D and is still able to reconstruct a solid object with good continuity; the 3D reconstruction result is presented in the Fig. 7.

### Laser scanning microscopic images of the drosophila brain

**Figure 8:**
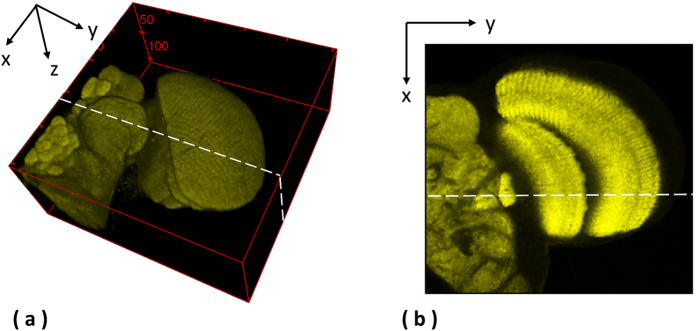
Extraction of side views for the laser scanning microscopic images of the drosophila brain^2^. One plane is randomly selected for the laser scanning microscopic images of the drosophila brain to extract the side views of the 3D reconstructed objects generated by individual methods.

**Figure 9:**
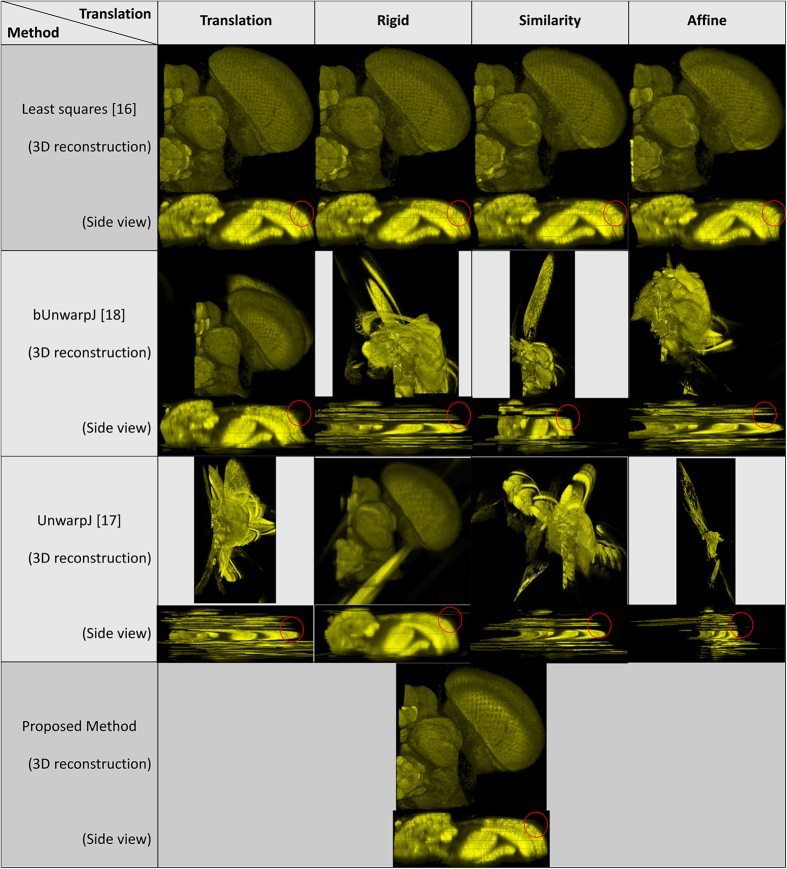
3D reconstruction results with associated side views for the laser scanning microscopic images^2^. 3D reconstruction results with associated side views of the 3D reconstructed objects generated by individual methods are presented for the laser scanning microscopic images of the drosophila brain^2^.

108 serial-section images of laser scanning microscopy of the drosophila brain is adopted as the fourth test set. This dataset is originally used to evaluate a 4D registration technology - BrainAligner by Peng *et al.*^2^, which aligns pairs of 3D brain volumes. As shown in Fig. 8, a plane is randomly defined to extract side views of reconstructed anatomical objected by individual methods, and 3D reconstruction results with associated side views are presented in Fig. 9. Some of the benchmark approaches fail to produce solid 3D objects, and the fourth benchmark approach^1^ fails to find corresponding features among sections and is not able to generate registration outputs. In comparison, the presented approach aligns images well in 3D and produces a solid object.

### Quantitative evaluation on a synthetic image sequence

**Figure 10:**
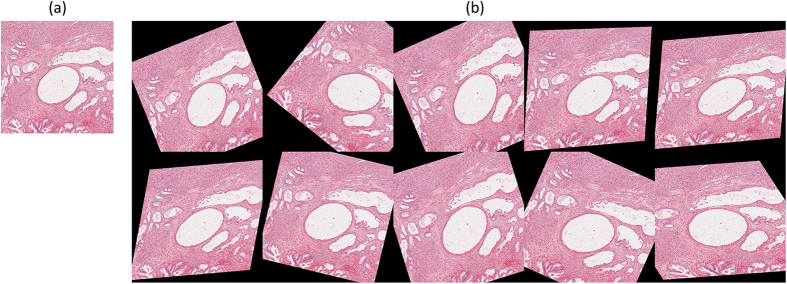
Synthetic image sequence for quantitative evaluation. The synthetic image sequence is build by duplicating multiple biological tissue images as multiple sections and then applying random deformation effects to individual layers. (**a**) The tissue image to be duplicated. (**b**) The synthetic image sequence containing ten randomly deformed tissue images.

For quantitative evaluation, a synthetic serial section test case is built by firstly creating a serial of ten identical images and secondly adding random deformation effects to individual images as shown in the Fig. 10. For the synthetic test case, a general registration performance measurement method, i.e. the percentage of pixels with similar intensity levels, is adopted to measure the registration accuracy, and an automatic evaluation tool is built to conduct quantitative evaluation automatically. Given a serial of images, 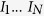, the overall registration accuracy, *R*, is formulated as the mean of the registration accuracies, *r*_*i*_, of individual pairs, 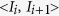.


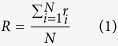



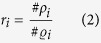


where 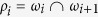 and 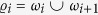; 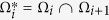 defines a mask common to the foreground Ω_*i*_ and Ω_*i*+1_ of *I*_*i*_ and *I*_*i*+1_; 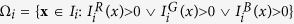 defines the foreground data after deformation, excluding the black background as shown in Fig. 10; 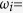 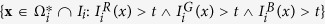 defines the white pixels in the common area 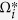 of *I*_*i*_; *t* = 200 in our experiments.

**Figure 11:**
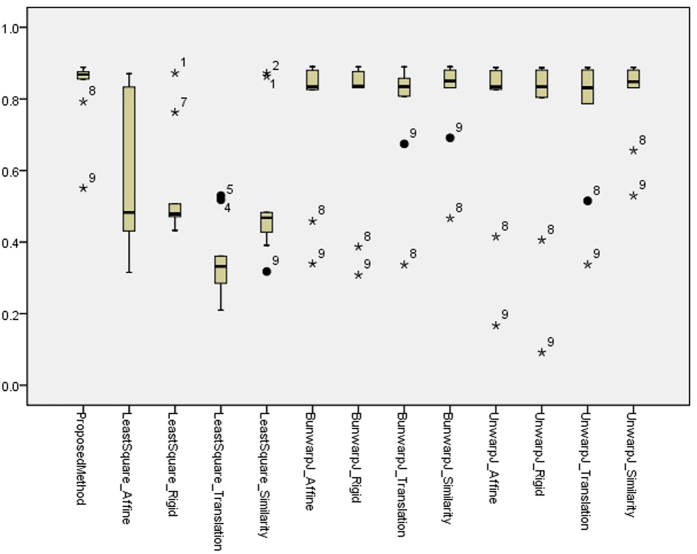
A box plot of the quantitative evaluation results on the synthetic image sequence. The presented methods works constantly well overall and outperforms the benchmark approaches. Outliers greater than 1.5× interquartile range (IQR) are marked with a dot, and outliers greater than 3 × IQR are marked with a asterisk.

**Figure 12:**
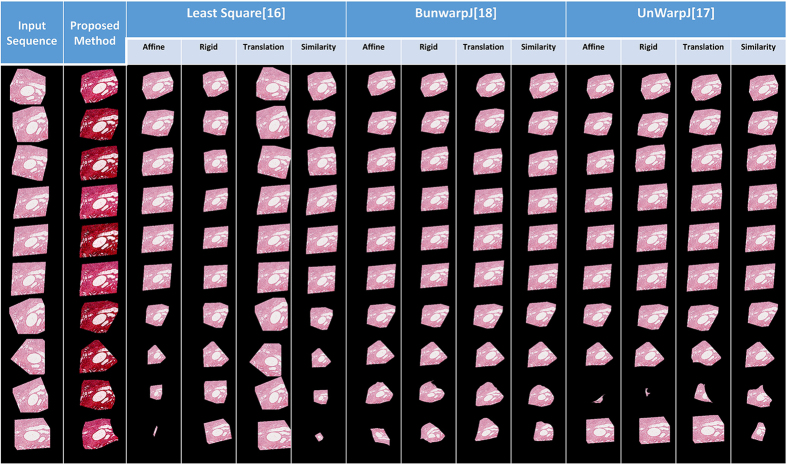
Image Inputs and Registration outputs for the synthetic image sequence. This figure shows the registration inputs and outputs by all methods for the synthetic test case.

Table 1 presents the quantitative evaluation results, and the box plot of the quantitative evaluation results is provided in Fig. 11. Figure 12 presents the registration outputs of individual approaches. The experimental results shows that the proposed method achieves higher registration accuracy score and performs better than the benchmark approaches.

## Discussion

We have presented a robust and fully automatic 3D image registration technique for detailed anatomical reconstruction of serial-section microscopic images. The method is promising to reassemble continuous volumes and able to deal with complex distortions, staining variations and artifacts. We have demonstrated our method in application to four different microscopic image sets, including two serial ssTEM images, one laser scanning confocal image sequences and one serial histopathological images. The registration and reconstruction results show that the proposed 3D image registration method is robust and performs consistently well, even for data sets with large morphological distortion problems. The presented 3D image registration technique is not limited to tissue images but can also be applied to other anatomically or histologically defined medical data and will prove to be a substantial advantage for any application that requires 3D image registration. The software implementation of the presented method and the test data used in this study are made publicly available for scientific communities to use (http://www-o.ntust.edu.tw/~cweiwang/3DRegistration/).

## Methods

**Figure 13:**
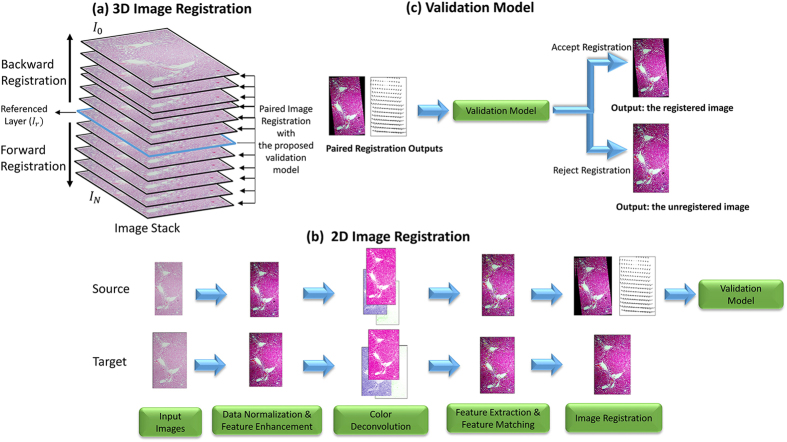
System framework of the proposed method. Given the referenced layer *I*_*r*_ specified 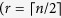 in this study), (**a**) the proposed 3D registration conducts forward and backward image registration sequentially and bidirectionally for every two neighboring image pairs. (**b**) The paired image registration consists of four steps: data normalization and feature enhancement, color deconvolution, feature matching and extraction, and image registration by using improved bi-directional elastic b-spline model. After the paired image registration is conducted to obtain an registered image with the associated deformation field, (**c**) a validation model is applied by evaluating the deformation field. If accepted, the paired image registration output is as the final 3D registration result. Otherwise, the original image is used as the registration output.

The proposed 3D image registration system combines and extends our recent efforts on robust 2D image registration^4^,^5^ and is devised with a new 3D alignment validation model utilizing the B-Spline deformation fields. The flowchart of the proposed method is shown in Fig. 13. Given the referenced layer *I*_*r*_ specified 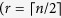 in this study), the proposed 3D registration conducts forward and backward image registration sequentially and bidirectionally for every two neighboring image pairs. The paired image registration consists of four steps: data normalization and feature enhancement, color deconvolution, feature matching and extraction, and image registration by using improved bi-directional elastic b-spline model. After the paired image registration is conducted to obtain an registered image with the associated deformation field, a validation model is applied by evaluating the deformation field. If accepted, the paired image registration output is as the final 3D registration result. Otherwise, the original image is used as the registration output. More details about each method can be found in the following subsections.

### Data Normalization and Feature Extraction

The data normalization process is applied to reduce variations on image features and enhance tissue patterns. This greatly benefits global feature matching and local area-based directing matching processes as it automatically adjust the brightness and contrast of image color channels based on the image histogram distribution.

In color images, the value of each pixel is represented by a vector 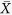 with elements the pixel values of each color component. Assuming 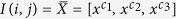 a random vector, which models the pixel value for each color component 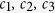 in a color image. To exclude extreme pixel values, which may not be representative of the main image content, we saturate a fixed percentage 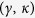 at the upper and lower ends of the target intensity range where 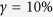 and 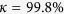 are used in this work. The lower and the upper bound intensity levels of the histogram of each channel, *x*_*low*_ and *x*_*high*_, are computed by the equations below. Given a histogram distribution *H*, where *H*(*x*) is the number of pixels with intensity level *x*, the lower and the upper bound values for transformation are formulated as follows.


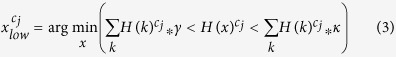



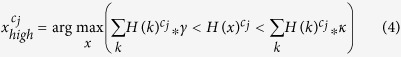


where 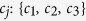.

Next, it maps the original pixel value 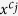 in the range from 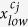 to 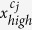 to new value 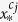 in the valid intensity scale from 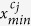 to 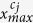.


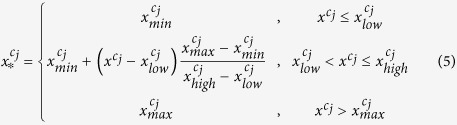


The data normalization is proven to reduce stain variation and enhance tissue patterns, furthermore improving the following feature extraction model accuracy to identify valid corresponding control points^4^.

Our goal is to extract the eosinophilic structures, which are generally composed of intracellular or extracellular protein, as image features for image registration, and the color decomposition technique is utilized to extract independent haematoxylin and eosin stain contributions from individual histopathological images using orthonormal transformation of RGB.

In the RGB color-space, every color is defined as 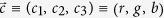 where *r*, *g*, *b* represent the red, green and blue components, and we can see additive color mixing as the vector addition of RGB components. To model the colors in an image as the vector addition of a desired (D) and undesired (U) components to a background color (P), new unit vectors can be defined as follows.


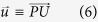



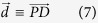



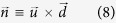


where 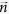 is perpendicular to 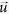 and 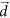; 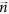, 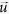, 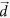 span the 3D space; 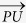 and 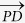 are alternative unit vectors based on the undesired and desired colors.

Then, color 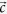 can be transformed to the new unit vectors.


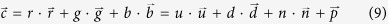


where 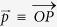; 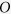 is the origin in the RGB 3D space; 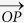 is a vector.

By setting *u* = 0, we remove the undesired component and obtain the new color 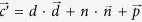. In the case of three channels, the color system can be described as a matrix of the form with every row representing a specific stain and every column representing the optical density (OD) as detected by the red, green and blue channel for each stain.


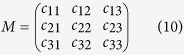


For normalization, each OD vector is divided by its total length, such that (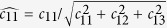, 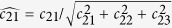 and 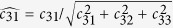). In this study, the normalized optical density (OD) matrix, 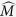, to describe the color system for orthonormal transformation is defined as follows:


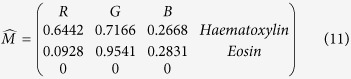


when C is the 3 × 1 vector for amounts of the stains at a particular pixel, the vector of OD levels detected at that pixel is equal to 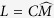. Therefore, multiplication of the OD image with the inverse of OD matrix results in orthogonal representation of the stains forming the image 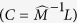. Then, the image features of the red channel are extracted as eosinophilic structures for both high level feature-based coarse registration and local area-based direct matching registration.

### 2D image registration

Our proposed 3D image registration framework is based on our previous work,2D robust image registration^4^,^5^. In order to improve robustness, Our 2D image registration method incorporate two approaches: area-based and feature-based. Initially, sparse approximation for fast and coarse global registration is applied. Given *I*_1_ and *I*_2_ as two images for alignment, *T* as a set of all possible transformation between *I*_1_ and *I*_2_ and *U*_*t*_(*I*) as the function that maps an image I to its transferred images using the transformation *t*, the goal is to find optimal transformation 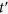
5:


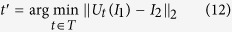


The transformation invariant distance 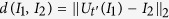 corresponds to the regular Euclidean distance when the images are aligned optimally in *L*^2^ where images are considered as continuous functions in 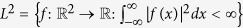, but finding the optimal transformation 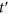 and the smallest distance *d*(*I*_1_, *I*_2_) is not easy as the objective function is non convex and local minima trap solution might occur. Feature based approaches represent a more efficient class of methods. Considered images can be well approximated by the sparse expansion in a series of geometric functions, we define 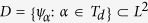 as a set of geometric features constructed by transforming a generating function 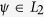 where 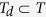 represents a finite discretization of the transformations *T* and 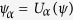 denotes the transformation of the generating function *ψ* by *α*. Given **p** and **q** as the respective K-sparse approximation of *I*_1_ and *I*_2_ in *D*,


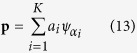



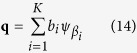


where *a*_*i*_, *b*_*i*_ are non-negative coefficients.

Then, the coarse global image registration problem can be formulated as finding the optimal relative transformation 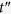 between the K-sparse approximations with the smallest approximate transformation invariant distance 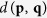:


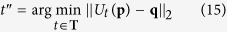



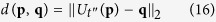


By utilizing the K-sparse approximations **p** and **q** are obtained by the previous procedures, and normalized image features **F**_1_, **F**_2_ from data normalization and feature extraction method, interested points **S**_1_, **S**_2_ can be detected by using the difference of Gaussian detector^19^ and then the corresponding feature points **p**, **q** are selected as geometric consensus between **S**_1_ and **S**_2_ using random sample consensus (RANSAC)^20^. The selected paired feature points **p**, **q** are then used for coarse global registration.

After alignment outputs are obtained from the coarse global registration process, they will be refined by area-based direct-matching method which is adapted from the improved bi-directional elastic b-spline model^18^. The registration methodology is based on the minimization of an energy function that incorporates four energy terms:


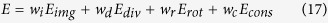


where *E*_*img*_ is the energy of the similarity error between *I*_1_ and 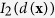, *E*_*div*_, *E*_*rot*_ are the regularization energy based on the divergence and curl of the deformation, *E*_*cons*_ expresses the geometrical consistency between the elastic deformation in both direction 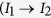, 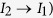, and *w*_*k*_ are the weights for sub-energy terms).

### 3D Image Registration

In the proposed 3D image registration framework, 2D image registration is sequentially and bidirectionally conducted for every image with the neighboring sections. Given 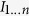 as a set of images to be aligned, 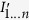 as the set of registered images after 3D registration, *I*_*r*_ as the referenced image 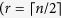 in this study), and *Reg* (*S*, *T*) as the 2D image registration function with *S* as the source image and *T* as the target image, a 3D image registration framework can be formulated as follow:


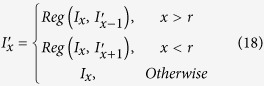


The aforementioned simple 3D framework however suffers from accumulated transformation errors or boosted over-deformation problems. When there are limited number of corresponding features detected or incorrect feature matching occurs, minor transformation error may be generated, and the transformation errors can be accumulated during the sequential registration process, causing over-deformed transformation results. Hence, in order to avoid over-deformation and accumulated transformation errors, we develop a validation model for the 3D registration framework. For every registration *Reg* (*S*, *T*), the validation model will automatically check if the transformation is valid or over-deformed. If valid, the 3D registration system will accept the transformation result 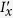 as the 3D registration output 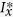.

### A validation model using deformation fields for 3D registration

During image registration, deformation fields are produced to represent the geometrical distances generated by the transformation function. Mathematically, a forward deformation function can be defined as follows.


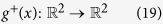


which means that this function transforms a two dimensional source image *I*_*s*_, into an image as similar as possible to the two dimensional target image *I*_*t*_. Here, the deformation fields are represented as a linear combination of B-splines, which can be formulated as follows.


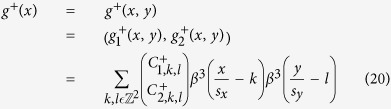


where *β*^3^ is the B-Spline to the third degree, *C*_*k*,*l*_ are the B-Spline coefficients, and *s*_*x*_ and *s*_*y*_ are scalars that control the degree of detail of the representation of the deformation field.

A validation model is designed to automatically compute the average of geometrical distances using deformation fields. The system will reject the registration output, 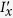, when the average of geometrical distances is too large, which indicates that the transformation is over-deformed.


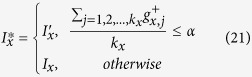


where *α* is a user-defined parameter that specifies the maximum of the mean deformation value to be allowed. Higher *α* value allows larger transformation but also allow higher accumulated transformation error, and hence over-deformations may occur with high *α* value. On the other hand, low *α* value may cause the validation model to reject a well-transformed registration output. Therefore, the *α* value should be low enough to reject over-deformed registration result and high enough to accept well-deformed registration. Table 2 shows the valid value range of the *α* value for individual datasets.

**Figure 14:**
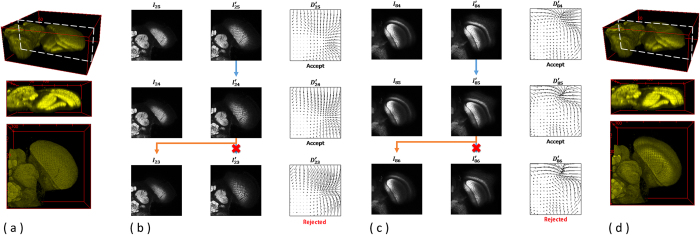
3D image registration with the presented validation model. Using the serial-section laser scanning microscope images of the drosophila brain data as example, (**a**) 3D reconstruction results with a side view of the raw input data are displayed; (**b**) an example of the backward registration from the image layer *I*_25_ to the image layer *I*_23_ is presented with associated deformation fields and validation processes. As the validation model accepts the deformation fields, 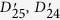, the outputs of the registration will be 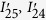. On the other hand, 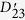 is rejected by the validation model, and the registration output will be *I*_23_. (**c**) Similarly, an example of the forward registration from Layer *I*_84_ to the image layer *I*_86_ is shown with associated deformation fields and validation processes. (**d**) 3D reconstruction results with a side view of the registered outputs are presented.

An illustration is given in Fig. 14 using the serial-section laser scanning microscope images of the drosophila brain data. (a) 3D reconstruction results with a side view of the raw input data are displayed; (b) an example of the backward registration from the image layer *I*_25_ to the image layer *I*_23_ is presented with associated deformation fields and validation processes. As the validation model accepts the deformation fields, 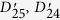, the outputs of the registration will be 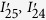. On the other hand, 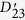 is rejected by the validation model, and the registration output will be *I*_23_. (c) Similarly, an example of the forward registration from Layer *I*_84_ to the image layer *I*_86_ is shown with associated deformation fields and validation processes. (d) 3D reconstruction results with a side view of the registered outputs are presented.

**Figure 15:**
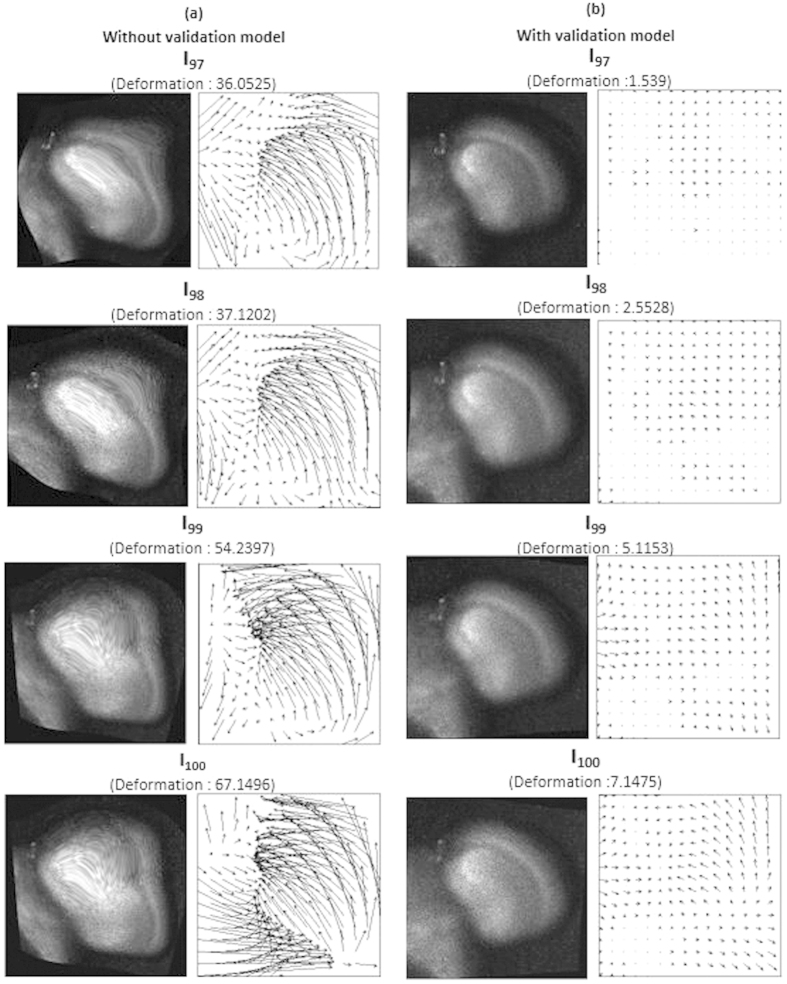
Compare the registration outputs with and without the validation model. This figure compares the registration outputs (**a**) without the validation model and (**b**) with the validation model applied, showing that the registration results without the presented validation model suffers from over-deformation problems and accumulates transformation errors. Over-transformed registration outputs accumulate the transformation errors, causing higher transformation error in the registration process of the next layer.

The validation model ensures that the accumulated over-deformation error does not proceed and affect the rest of the registration process. Figure 15 compares the registration results with associated deformation fields with and without the proposed validation model, showing that the registration results without the presented validation model suffers from over-deformation problems and accumulates transformation errors. Over-transformed registration outputs accumulate the transformation errors, causing higher transformation error in the registration process of the next layer.

### Data and Software

The software implementation of the presented method is developed in JAVA (with jdk 1.7.0.51 installed) and based on Fiji framework^16^ and TrakEM2 image registration framework^9^. The software and the data are both made publicly available for scientific communities to use (http://www-o.ntust.edu.tw/~cweiwang/3Dregistration/).

## Additional Information

**How to cite this article**: Wang, C.-W. *et al.* Fully automatic and robust 3D registration of serial-section microscopic images. *Sci. Rep.* **5**, 15051; doi: 10.1038/srep15051 (2015).

## Figures and Tables

**Table 1:** Quantitative evaluation on the synthetic test case.

Method		Registration Accuracy (*R*)
Proposed Method		0.8268
Least Square^16^	Affine	0.6075
	Rigid	0.546
	Translation	0.347
	Similarity	0.5249
BunwarpJ^18^	Affine	0.756
	Rigid	0.7438
	Translation	0.7735
	Similarity	0.7978
UnwarpJ^17^	Affine	0.7299
	Rigid	0.7199
	Translation	0.7511
	Similarity	0.8026

^*^Elastic method^1^ fails in producing registration outputs.

**Table 2:** *α* value range for each dataset.

Dataset	*α* value range
ssTEM Drosophila melanogaster third instar larva VNC^14^	20–200
ssTEM Drosophila melanogaster first instar larva VNC^9^	30–95
Serial histopathological images of renal cortical tissues^4^	200–350
Laser scanning microscopic images of the drosophila brain^2^	10–30
